# Assessing the association between a postpartum intrauterine device intervention and perceived pressure to adopt contraception in Sri Lanka^☆^

**DOI:** 10.1016/j.contraception.2025.111242

**Published:** 2025-10-01

**Authors:** Brooke W. Bullington, Katherine Tumlinson, Leigh Senderowicz, Joanna Maselko, Kavita Shah Arora, Jessie K. Edwards, Audrey Pettifor

**Affiliations:** a Department of Epidemiology, Gillings School of Global Public Health, University of North Carolina, Chapel Hill, NC, United States; b Division of Family Planning, Department of Obstetrics and Gynecology, University of Utah, Salt Lake City, UT, United States; c Carolina Population Center, University of North Carolina, Chapel Hill, NC, United States; d Department of Health Policy and Management, Gillings School of Global Public Health, University of North Carolina, Chapel Hill, NC, United States; e Department of Gender and Women’s Studies, University of Wisconsin, Madison, WI, United States; f Department of Obstetrics and Gynecology, University of Wisconsin, Madison, WI, United States; g Department of Obstetrics and Gynecology, University of North Carolina, Chapel Hill, NC, United States

**Keywords:** Autonomy, Choice, Intrauterine device (IUD), Long-acting reversible contraception (LARC), Postpartum

## Abstract

**Objective(s):**

Traditional measures of success in family planning research focus on contraceptive uptake and use. Scholars have increasingly challenged these outcomes, calling for rights-based, person-centered measures that capture whether individuals can fulfill their reproductive preferences or desires. We sought to evaluate a family planning program using a novel, person-centered measure of perceived pressure to adopt contraception.

**Study design:**

We use secondary data collected as part of the Postpartum Intrauterine Device (PPIUD) Study, a step-wedged, cluster-randomized trial of a provider-focused PPIUD intervention conducted in Sri Lanka from 2015 to 2018. We used logistic regression to assess the association between the PPIUD intervention and perceived pressure to adopt contraception at 18-month follow-up and converted estimates into risk differences.

**Results:**

About 18 months following the PPIUD intervention, 13% of participants were not using contraception, most (82%) were using contraception and did not report feeling pressured to adopt their method, and 5% were using contraception and did report feeling pressured. While the PPIUD intervention was associated with increased contraceptive use, it was also associated with increased perceived pressure to adopt contraception (risk difference comparing intervention and control group: 4.5%; 95% confidence interval: −2.7%, 11.7%). Perceived pressure to adopt contraception was higher among IUD users compared to users of other contraceptive methods.

**Conclusion:**

While expanding access to contraceptive methods – including the IUD – is imperative to promote choice in contraceptive decision-making, our findings highlight the importance of evaluating interventions not just based on use or uptake of specific contraceptive methods, but also using person-centered outcomes that capture reproductive autonomy.

**Implications:**

Evaluating family planning programs solely on contraceptive use overlooks critical aspects of reproductive autonomy. We find that a postpartum IUD intervention in Sri Lanka was associated with increased contraceptive use but also increased perceived pressure to adopt contraception. Our findings emphasize the importance of assessing programs using rights-based, person-centered measures.

## 1. Introduction

Researchers have historically evaluated global family planning programs using measures of contraceptive prevalence [1–4]. Programs are deemed successful if the proportion of participants using contraception or using a specific method of contraception increases following an intervention. In recent years, scholars have challenged outcome measures focused on contraceptive use [5–7], noting that they were originally designed as a means for promoting fertility reduction during the population control era [6]. As a result, evaluating a program based primarily on contraceptive use inherently assumes that contraceptive use – or use of a specific contraceptive method – is beneficial for all people with the capacity to get pregnant, regardless of their contraceptive preferences [5,6]. Researchers have therefore sought to develop new person-centered, population-based measures of success for family planning programs [5,6,8], though the field still lacks standardized outcomes that center individuals’ contraceptive preferences [9].

The Postpartum Intrauterine Device (PPIUD) Study serves as a case study for highlighting the potential pitfalls of evaluating an intervention based on contraceptive uptake. The study, which aimed to institutionalize PPIUD services in multiple low- and middle-income countries (LMICs), was initially evaluated based on PPIUD uptake [10]. Researchers found that the intervention, which included provider training in PPIUD counseling and insertion, was associated with increased PPIUD counseling, modern contraceptive use, and PPIUD use in Tanzania, Sri Lanka, and Nepal [11–13]. However, additional analyses examining the PPIUD intervention in Tanzania also revealed that the intervention was associated with biased contraceptive counseling, with providers emphasizing the IUD and de-emphasizing or not mentioning other contraceptive methods [14]; people delivering babies at hospitals with the PPIUD intervention were more likely to be counseled on the IUD alone and less likely to be counseled on other methods [15]. While the PPIUD study in Tanzania increased PPIUD uptake, it also decreased one important aspect of contraceptive autonomy: informed contraceptive choice, or the ability for participants to make decisions about contraceptive use based on unbiased information about a range of contraceptive options [6].

Free contraceptive choice – or whether a person can make decisions about contraception voluntarily, free from barriers and coercion – is a vital aspect of contraceptive autonomy that typically goes unmeasured in the evaluation of family planning programs [6]. Prior studies in LMICs have reported numerous violations to free choice, including individuals feeling encouraged, pressured, or forced to use contraception when they do not desire a method [16–19] or not being able to access removal services for long-acting reversible contraception [20–23]. Capturing such experiences is imperative for understanding whether programs achieve their goals and ensuring that they do not violate critical aspects of human rights and reproductive autonomy.

The objective of this manuscript is to evaluate whether individuals in the PPIUD Study, described above, felt pressured to adopt contraception based on a person-centered measure of free contraceptive choice, using data from Sri Lanka. While prior research has found that the PPIUD intervention in Sri Lanka increased PPIUD uptake [11], we seek to understand whether the intervention may have led participants to feel pressured to adopt contraception.

## 2. Materials and methods

### 2.1. Background

This is a secondary analysis of data collected from the Postpartum Intrauterine Device (PPIUD Study), a stepped wedged, cluster-randomized trial of a PPIUD intervention conducted in Sri Lanka, Tanzania, and Nepal from 2015 to 2018. This is a secondary analysis of data collected in Sri Lanka. In the other countries, the primary outcome of this analysis was either not measured (Nepal) or could not be constructed due to a skip pattern (Tanzania).

The PPIUD intervention, which has been described in-depth previously [10], was developed by the International Federation of Gynecology and Obstetrics (FIGO) and implemented by the Sri Lanka College of Obstetricians and Gynecologists. The intervention was delivered at the hospital level and involved clinician training, including classroom-based learning, (i.e., lectures, videos, and web-based learning) and on-the-job teaching and mentoring (i.e., supervised IUD insertions and contraceptive counseling sessions). Women visiting hospitals with the PPIUD intervention received information about postpartum contraception in general and the availability of PPIUD services, including PPIUD counseling and exposure to information about PPIUDs.

Six hospitals in Sri Lanka were selected for inclusion based on geography. Hospitals were then matched into pairs based on their annual number of deliveries. One hospital from each pair was randomly assigned to the PPIUD intervention. Hospitals assigned to the intervention received the hospital standard-of-contraceptive care for the three months followed by the PPIUD intervention for 15 months. The other facility in each pair received the standard-of-care for nine months followed by the PPIUD for 9 months.

Women who delivered a baby at an included hospital during the study period and resided in Sri Lanka were invited to participate in the study. Women who agreed to participate in the study completed a baseline survey and up to three follow-up surveys.

The Ethics Review Committee at the Faculty of Medicine at the University of Colombo in Sri Lanka provided ethical approval for this study. The Institutional Review Boards at Harvard University and University of North Carolina at Chapel Hill deemed this study exempt, as all data was de-identified.

### 2.2. Data collection and analytic sample

The study team initially developed baseline and follow-up surveys in English, which were translated into local languages. Surveys contained questions about demographic characteristics, contraceptive counseling experiences, PPIUD-specific counseling, and PPIUD insertion. Surveys were pilot tested and updated based on feedback.

Trained female enumerators administered baseline surveys to eligible women in postnatal hospital wards following childbirth and PPIUD insertion (if applicable) but prior to hospital discharge. Women completed follow-up surveys in person at study hospitals, their homes, or satellite clinics.

Participants who adopted PPIUDs were invited to participate in three follow-up visits at 2-4 months, nine months, and 18 months postpartum. A random sample of participants who did not adopt PPIUDs were selected to participate in 9- or 18-month follow-up. We accounted for this sampling approach using sampling weights, described in detail below.

### 2.3. Measures

The primary exposure is a binary indicator of whether a participant gave birth in a hospital that had or had not yet implemented the PPIUD intervention.

The primary outcome was captured only at 18-month follow-up and was determined based on: (a) whether a participant was using contraception and (b) among those using contraception, whether or not they felt pressured to use their current method. We operationalized whether a participant felt pressured to adopt contraception using the survey question: “Did you feel pressured into using [your method]?” Contraceptive non-users were not asked if they have experienced pressure to use contraception. All participants were categorized as either: (1) not using contraception, (2) using contraception, did not feel pressured, or (3) using contraception, did feel pressured. We refer to this three-level outcome measures as perceived pressure to adopt contraception. We implemented a three-level outcome measure among all participants rather than a two-level outcome only among current contraceptive users because contraceptive use is likely influenced by the exposure (the PPIUD intervention) and is therefore a collider; conditioning on contraceptive use could induce collider bias, a form of selection bias that occurs when controlling for a collider.

### 2.4. Weights

All participants who adopted PPIUD after delivery and a random sample of participants who did not were selected for 18-month follow-up. To account for this sampling strategy, we developed sampling weights. We used logistic regression to estimate the probability that an individual was selected for 18-month follow-up conditional on whether they adopted a PPIUD. All participants who were selected for 18-month follow-up were assigned a sampling weight, which was the inverse probability of being selected for 18-month follow-up conditional on PPIUD adoption.

We also created inverse probability of observation weights (IPOW) to account for missingness among participants who were selected for 18-month follow-up but missing outcome data. The 18-month follow-up visit was considered missing if a participant selected for 18-month follow-up either: (1) did not complete the visit (*n* = 1712, 17% of those selected for follow-up) or (2) was missing data on the outcome (*n* = 650, 6% of those selected for follow-up), for a total of 2362 missing records (*n* = 22% of those selected for follow-up). We used a causal diagram to identify baseline characteristics needed for d-separation – or conditional independence, given a set of other variables – between missingness and the outcome; characteristics included treatment group, age, parity, education level, hospital of delivery, and PPIUD insertion after delivery. We used logistic regression to estimate the probability that an individual completed 18-month follow-up conditional on the aforementioned covariates. The IPOW for a participant who completed 18-month follow-up was the inverse probability of completing that visit, conditional on covariates.

We created our final survey weights by multiplying sampling weights by IPOW. We present weighted results for all analyses.

### 2.5. Statistical analysis

We described the sociodemographic characteristics of the sample and the distribution of the primary outcome by PPIUD intervention status and participants characteristics (age, parity, education level). We used Chi-square tests to compare the distribution of the outcome by sociodemographic characteristics. We ran multinomial logistic regression with robust standard errors accounting for clustering by facility and using contraception and not feeling pressured as the referent category to estimate the association between the PPIUD intervention and whether participants had perceived pressure to adopt contraception at 18-month follow-up. Because the PPIUD intervention was randomized to facilities, women who received counseling at PPIUD intervention and control facilities were exchangeable by design; we verified exchangeability by examining balance in key variables between intervention and control arms. Thus, we did not control for covariates. We used model outputs to calculate risk differences and employed the Delta Method to construct 95% confidence intervals [24]. Finally, we limited our sample to current contraceptive users. Among participants who reported using a contraceptive method that requires an interaction with a provider (including female permanent contraception, IUD, injectable, implant, and contraceptive pill), we examined the proportion who had perceived pressured to adopt contraception by current method at 18-month follow-up. All statistical analyses were conducted in SAS Software version 9.4 (SAS Inc, Cary, NC).

## 3. Results

A total of 40,504 participants completed the baseline survey and 10,631 were selected for 18-month follow-up. About 60% of participants delivered at hospitals with the PPIUD intervention, whereas about 40% delivered at hospitals without the intervention. Participant characteristics were similar among participants who delivered at hospitals with and without the PPIUD intervention (Table 1). Most participants were aged 21–35, and nearly all were married or living with their partner. The majority of participants had a parity of one (38.6%) or two (37.6%). Just under a third of participants had completed secondary school (31.7%) and just over half had attended more than secondary school (50.6%). About 6.8% of participants adopted a PPIUD after delivery.

In Table 2, we show the proportion of participants who reported using contraception and feeling pressured by sociodemographic characteristics; younger (< 21 years) and older (36+ years) participants, participants with higher parity, and participants with less education were significantly more likely to report using contraception and feeling pressured (*p* < 0.001 for all).

We present the distribution of the primary outcome, perceived pressured to adopt contraception, by PPIUD intervention status in Table 3. About 13.4% of the sample reported not using contraception, with a lower proportion of participants who delivered at hospitals with compared to without the PPIUD intervention reporting not using contraception (12.1% vs. 16.9%). Most participants reported using contraception and not feeling pressured (81.8%), and this did not differ by intervention status (81.9% vs. 81.6%). However, a higher proportion of participants who delivered at PPIUD intervention compared to control hospitals reported using contraception and feeling pressured (6.1% vs. 1.6%, 4.8% in the overall sample). The PPIUD intervention was therefore associated with increased contraceptive use at 18-month follow-up, but only among those who reported feeling pressured to use contraception.

In Table 4, we report risk differences and 95% confidence intervals for the association between the PPIUD intervention and perceived pressure to adopt contraception, with using contraception and not feeling pressured as the referent category. The 18-month risk of not using contraception was 4.8 percentage points lower among participants who delivered at hospitals with the PPIUD intervention compared without the PPIUD intervention (95% confidence interval: −6.2%, −3.4%); that is, a higher proportion of participants who delivered at hospitals with the PPIUD intervention were using contraception. The 18-month risk of using contraception and feeling pressured was 4.5 percentage points higher among those who delivered at hospitals with compared to without the PPIUD intervention (95% CI: −2.7%, 11.7%); that is, the PPIUD intervention was associated with increased risk of participants using contraception and feeling pressured, though the confidence interval crossed the null value. Thus, while the PPIUD intervention was associated with increased contraceptive use, the increased use was among participants who reported feeling pressured to adopt a method.

We examine whether contraceptive users experienced perceived pressured to adopt contraception by current method and intervention status at 18-month follow-up in Table 5. About 15.0% of contraceptive users reported using the IUD. Of IUD users, 10% reported feeling pressured to adopt contraception; a higher proportion of IUD users who delivered at intervention hospitals compared to control hospitals reported pressure to adopt contraception (11.6% vs. 5.0%). For all other contraceptive methods, the proportion of participants who reported feeling pressured to adopt contraception ranged from 3% (for the implant) to 6% (for female permanent contraception and the injectable). The proportion of users who reported feeling pressured to adopt contraception was higher among those who delivered at intervention compared to control hospitals for all methods except the implant.

## 4. Discussion

Following a postpartum IUD intervention in Sri Lanka, we find that participants who delivered at hospitals that received the intervention were more likely to report using contraception and feeling pressured to adopt their method compared to participants who delivered at hospitals without the intervention, though confidence intervals for the risk difference included the null value. Most participants reported using contraception and not feeling pressured. Younger participants, participants with higher parity, and participants with less education were more likely to report using contraception and feeling pressured. A higher proportion of IUD users reported pressure to adopt contraception compared to users of other methods, and a higher proportion of IUD users who delivered in intervention compared to control hospitals reported pressure to adopt the IUD. Our findings highlight the importance of evaluating interventions not just based on uptake or use of contraception or a specific contraceptive method, but also using person-centered outcome measures that capture whether participants are able to exercise autonomy in their contraceptive decision-making.

Our findings suggest that the PPIUD study in Sri Lanka was associated with increased contraceptive use at 18-month follow-up, part of this increased use was among participants who reported feeling pressured to adopt their method. If we evaluated the study exclusively based on traditional measures of programmatic success – whether the intervention increased contraceptive use – we would find that contraceptive use was approximately 5 percentage points higher in the intervention compared to control arm, and therefore might deem the program successful. However, our person-centered outcome demonstrates that the increase in contraceptive use in the intervention arm was only among those who felt pressured to use a method; there was no difference in the proportion of participants who were using contraception and did not feel pressured between the intervention and control arms. The stated goal of many family planning programs is to increase voluntary contraceptive use, or contraceptive use among those who desire contraception [25]. Yet, voluntary use of contraception cannot be determined without person-centered outcome measures that capture whether people can make informed, full, and free contraceptive choices [6].

Prior studies have evaluated the same PPIUD intervention in Tanzania and found that participants who delivered at hospitals with the intervention were more likely to be counseled on the IUD alone and less likely to be counseled on other methods [14,15], thereby inhibiting informed contraceptive choice, a key component of contraceptive autonomy [6]. The present study evaluates the same PPIUD intervention in a different context using a novel measure of free contraceptive choice, which we define as whether participants felt pressured to adopt contraception. In conjunction with this prior work, we posit that (1) the choice to focus on PPIUD uptake as a primary outcome measure and (2) implementing monitoring systems throughout the study that ensured study staff were aware of PPIUD uptake [26] may have inadvertently inhibited participant’s contraceptive autonomy. Expanding access to a wide range of contraceptive methods, including the IUD, is imperative; yet, as our findings demonstrate, focusing initiatives on a singular method without carefully consideration of potential consequences on informed and free contraceptive choice can have unintended consequences that inadvertently limit reproductive autonomy.

Few studies have evaluated family planning programs using measures of autonomy in contraceptive decision-making, and even fewer have attempted to measure free contraceptive choice, or whether people could make decisions about contraception free from coercion. Our study implements a novel measure of perceived pressure to adopt contraception. Strengths of this work include the large sample size and the cluster-randomized step-wedged study design. Our study also has several limitations. First, while our novel measure of perceived pressure to adopt contraception was informed based on qualitative data and pilot testing in Burkina Faso, we were not able to conduct cognitive interviewing around our outcome given limitations of using secondary data. Future work developing and assessing measures of free contraceptive choice, and implementing previously developed measures of agency in contraceptive decision-making (for example, the Contraceptive Agency Scale), is imperative for understanding the impacts of family planning interventions [27]. Additionally, perceived pressure to adopt contraception was measured only at 18-month follow-up. Participants may have experienced pressure to adopt contraception outside of the PPIUD intervention, though we hypothesize that such experiences of pressure would be evenly distributed in the intervention and control groups. Further, only those who were using contraception at 18-month follow-up were asked if they felt pressure to adopt their method. Participants who received contraceptive counseling but did not adopt a contraceptive method may also have felt pressure, but we do not capture such experiences with our measure. Loss-to-follow-up may have led to selection bias in our sample; we used inverse-probability of observation weights in attempt to mitigate such bias. Still, the probability of observation may have been related to an unmeasured variable that was not accounted for in our weights model.

Our findings shed light on the importance of measuring and evaluating family planning programs using rights-based, person-centered outcomes that capture quality of contraceptive care and autonomy in contraceptive decision-making. Scholars have proposed and developed new measures of success for global family planning programs [5,6,8,16,28,29]; through this work, they have also discussed the difficulty of capturing nuanced experiences, like violations of reproductive autonomy, on population-based surveys [16,29]. The present study underlines the importance of continuing to develop, refine, and expand such measures to allow researchers and program implementers to understand how family planning interventions may impact a wide array of person-centered outcomes. Without measures of agency and autonomy, family planning programs may continue to have unintended consequences that negatively impact people’s reproductive lives.

## Figures and Tables

**Table 1:** Unweighted^a^ baseline characteristics of PPIUD study participants in Sri Lanka, 2015–2018

	PPIUD intervention	No PPIUD intervention	Total
Unweighted *n*	23,666	16,838	40,504
Age (years)			
< 21	1968 (8.3%)	1468 (8.7%)	3436 (8.5%)
21–25	6153 (26.0%)	4323 (25.7%)	10,476 (25.9%)
26–30	7770 (32.8%)	5508 (32.7%)	13,278 (32.8%)
31–35	5518 (23.3%)	3941 (23.4%)	9459 (23.4%)
36+	2257 (9.5%)	1598 (9.5%)	3855 (9.5%)
Marital status			
Married or living with partner	23,621 (99.8%)	16,793 (99.7%)	40,414 (99.8%)
Not married or living with partner	45 (0.2%)	45 (0.3%)	90 (0.2%)
Parity (births)			
1	9263 (39.1%)	6386 (37.9%)	15,649 (38.6%)
2	8949 (37.8%)	6269 (37.2%)	15,218 (37.6%)
3+	5454 (23.1%)	4183 (24.8%)	9637 (23.8%)
Education level			
Completed primary school or less	969 (4.1%)	732 (4.4%)	1701 (4.2%)
Some secondary school	3134 (13.2%)	2329 (13.8%)	5463 (13.5%)
Completed secondary school	7695 (32.5%)	5146 (30.6%)	12,841 (31.7%)
More than secondary school	11,868 (50.2%)	8631 (51.3%)	20,499 (50.6%)
PPIUD inserted	2169 (9.2%)	584 (3.5%)	2753 (6.8%)

PPIUD, Postpartum Intrauterine Device.

a Data reported in this table were collected at baseline and were complete for all participants. Therefore, sampling weights and inverse probability of observation weights were not applied.

**Table 2:** Weighted proportion^a^ of participants who reported using contraception and feeling pressure, by sociodemographic characteristics (unweighted *n* = 8290, weighted *N* = 40,443), Sri Lanka, 2015–2018

	Proportion who reported using contraception, felt pressured	*p*-value
Total	4.8%	
Age (years)		< 0.001
< 21	6.4%	
21–25	4.5%	
26–30	4.7%	
31–35	4.4%	
36+	5.4%	
Parity (births)		< 0.001
1	4.6%	
2	4.5%	
3+	5.5%	
Education level		< 0.001
Completed primary or less	14.4%	
Some secondary	9.6%	
Completed secondary	3.4%	
More than secondary	4.0%	

a Proportions were calculated by dividing the number of individuals in a given category (ie. aged < 21 years) who reported using contraception and feeling pressure to adopt it by the total number of individuals in that category.

**Table 3:** Weighted^a^ perceived pressure to adopt contraception at 18-month follow-up^b^ and 95% confidence interval by PPIUD intervention group, Sri Lanka, 2015–2018 (unweighted *n* = 8290, weighted *N* = 40,443)

	PPIUD intervention	No PPIUD intervention	Total
Not using contraception *Weighted*%	12.1% (11.2%, 12.9%)	16.9% (15.2%, 18.5%)	13.4% (12.6%, 14.2%)
Using contraception, did not feel pressured *Weighted*%	81.9% (80.8%, 82.9%)	81.6% (79.9%, 83.3%)	81.8% (80.9%, 82.7%)
Using contraception, did feel pressured *Weighted*%	6.1% (5.5%, 6.7%)	1.6% (1.1%, 2.1%)	4.8% (4.3%, 5.2%)

PPIUD, Postpartum Intrauterine Device.

a We developed inverse probability of sampling weights and inverse probability of observation weights to account for selection into 18-month follow-up and loss-to-follow-up, respectively. The weighted *N* = 40,443.

b The survey question about whether a participant felt pressured to adopt contraception was only asked to current contraceptive users. Because the PPIUD intervention likely influenced contraceptive uptake, we used a three-level outcome variable, including those who were not currently contraception.

**Table 4:** Association between the PPIUD intervention and perceived pressure to adopt contraception at 18-month follow-up, weighted, Sri Lanka, 2015–2018, (unweighted *n* = 8290, weighted *N* = 40,443)

Outcome category	Risk difference^a^	95% confidence interval
Not using contraception	−4.8%	−5.9%, −3.7%
Using contraception, did not feel pressured	ref	-
Using contraception, did feel pressured	4.5%	−2.7%, 11.7%

PPIUD, Postpartum Intrauterine Device.

a Risk difference is comparing individuals who delivered at hospitals with the PPIUD intervention and individuals who delivered at hospitals without the PPIUD intervention. Estimates were derived from a weighted multinomial logistic regression model that account for clustering by facility with “using contraception, did not feel pressured” as the referent outcome.

**Table 5:** Weighted percent of current contraceptive users^a^ who reported pressure to adopt contraception by current contraceptive method^b^ at 18-month follow-up, stratified by intervention status, Sri Lanka, 2015–2018

Contraceptive method used	Weighted percent of contraceptive users^c^	Weighted percent of method users who report using the method and feeling pressured to adopt contraception		
		Total	PPIUD intervention	No PPIUD intervention
Female permanent contraception	8.4%	6.1% (4.3%, 8.0%)	7.4% (5.0%, 9.8%)	3.1% (0.6%, 5.5%)
Intrauterine device	15.0%	10.1% (8.8%, 11.5%)	11.6% (10.0%, 13.2%)	5.0% (2.8%, 7.3%)
Injectable	23.6%	5.9% (4.8%, 7.1%)	7.0% (5.7%, 8.4%)	1.5% (0.2%, 2.8%)
Implant	9.0%	3.3% (1.9%, 4.7%)	3.3% (1.7%, 4.9%)	3.3% (0.4%, 6.2%)
Pill	11.3%	3.5% (2.2%, 4.7%)	5.5% (3.5%, 7.4%)	0.0% (0.0%, 0.0%)

PPIUD, Postpartum Intrauterine Device.

a Only includes contraceptive users using methods that require an interaction with a provider.

b Participants could select multiple contraceptive methods.

c Weighted percent of contraceptive users are percentages of all contraceptive users. Given that we only present methods that require an interaction with a provider, percentages do not add to 100%.
